# Diminishment the gas permeability of polyethylene by “densification” of the amorphous regions

**DOI:** 10.1038/s41598-023-46276-9

**Published:** 2023-11-13

**Authors:** Marta Safandowska, Cezary Makarewicz, Artur Rozanski, Rafal Idczak

**Affiliations:** 1 Centre of Molecular and Macromolecular Studies, Polish Academy of Sciences, Sienkiewicza 112, 90-363 Lodz, Poland; 2 The Bio-Med-Chem Doctoral School of the University of Lodz and Lodz Institutes of the Polish Academy of Sciences, Banacha 12/16, 90-237 Lodz, Poland; 3 Institute of Experimental Physics, University of Wroclaw, Maksa Borna 9, 50-204 Wroclaw, Poland

**Keywords:** Engineering, Materials science

## Abstract

High-density polyethylene/paraffin wax (HDPE/wax) systems with adjustable density of the amorphous regions were prepared by a melt-blending process to optimize/control the final oxygen barrier properties. The introduction of paraffin wax (a low molecular weight modifier) is the key to tune the gas permeability properties of polyethylene-based materials. Density gradient column (DGC) measurements distinctly showed that the incorporation of modifier led to densification of the amorphous phase of semicrystalline HDPE consisting in a decrease in the average fractional free volume confirmed by positron annihilation lifetime spectroscopy (PALS). Polyethylene with “densified” amorphous phase exhibits lower oxygen permeability parameters compared to pristine polyethylene, but it is characterized by similar thermal and thermomechanical properties. An increase in the density of the amorphous regions of polyethylene by about 0.003 g/cm^3^, which corresponds to 0.3%, reduces the permeability of oxygen by up to 22%. For the first time, it has been proven that by controlling the density of the amorphous regions of semicrystalline polymers, it is possible to obtain materials with appropriate transport properties (without changing other properties) for applications meeting specific requirements.

## Introduction

Since the 1940s, plastics have successively replaced metals and glass in the packaging industry. Today, the annual global production of plastics is around 450 million tons, of which 40% are packaging^1, 2^. However, plastics are inherently permeable to many gases, therefore the growing demands on packaging products and the growing possibilities of their use make research into the development of new and improved barrier property plastics broadly escalated^1, 3^. Plastics, depending on the type of polymer, are known to exhibit varying barriers to gases and water vapor.

Polyethylene, one of the most widely used thermoplastic polymers, demonstrate for instance good water vapor but poor oxygen barrier properties. Taking into account the above and the growing environmental pollution with plastics originating from the packaging sector, which is currently estimated at approximately 154 million tons of waste generated annually worldwide^2^, instead of synthetic polymers derived from petrochemicals, biodegradable polymers made from renewable resources can be introduced^4–6^. Biodegradable polymers are, however, less processable, less thermally stable and still more expensive compared to petrochemical-based plastics. It is therefore worth considering strengthening the oxygen barrier properties of “classic” synthetic polymers commonly used in the packaging industry.

Polyethylene is widely used in packaging due to a good resistance to chemicals/harsh environments, non-toxicity and recyclability. Moreover, it meets the requirements of thermal stability for the melt-flow processes applied in most manufacturing applications.

To improve the barrier character of polyethylene to oxygen, different strategies have been identified^7–12^. Surface treatment (coating/lamination with gas barrier polymers)^13^, addition of oxygen scavengers^14^, and use of multilayer films^15^ are a few examples of the well-known/described methods. The barrier performance of polyethylene can be also influenced by adding impermeable additives/nanoadditivies to polymer matrix. However, the difficulty in obtaining adequate particle dispersion and the compatibility between the additive particles and the polymer have to be taken into account. The orientation of impermeable particles perpendicular to the diffusion direction can maximize their barrier effect^16, 17^. The strategies currently used to achieve these objectives (magnetically induced orientation^18^, electric field-induced orientation^19^, layer-by-layer assembly^11, 20^) unfortunately are not directly correlated with the classical polymer technological processes for large scale industrial production and therefore it is hard to envision their applicability in packaging systems.

Polyethylene is a representative semicrystalline polymer which, during the solidification process, demonstrate the ability to crystallize. The transport of gases and vapors in the case of semicrystalline polymers takes place only through the amorphous regions, the crystals are impermeable for even the smallest molecules of penetrant^21, 22^. Gas/vapor permeation through polymer material involves the absorption of permeating molecules into the polymer surface, solubility into and diffusion through the polymer and desorption from the other side^23, 24^. The gas molecules cannot diffuse and dissolve in the crystalline domain, thus crystalline zones are excluded volumes for permeants. The differences in barrier properties between the crystalline and amorphous phase stem mainly from different density of packing of macromolecules within these regions.

Keeping that in mind, the barrier properties of the material with a given crystalline microstructure mainly depend on the structure of the amorphous component. Due to its very complex and irregular structure (both chemically and physically), the rate of transport of the penetrator molecules (mainly by means of Brownian motions) will be dependent on the density of macromolecules packing, which determine the content of the free volume of the amorphous phase and the size of its pores^25, 26^.

The single experimental studies have shown that by removing or introducing non-polymeric substances from or into non-crystalline regions, it is possible to change the molecular packing of the amorphous phase, as well as control the size of the free volume, which determines the final barrier properties of the material without significant changes of the other physicochemical properties^27–29^.

Detailed understanding of the role of the nanostructure of amorphous phase regions in the barrier properties of semicrystalline polymers is a great importance both at fundamental and application level. This study aimed to obtain high density polyethylene/paraffin wax systems with improved barrier against the oxygen transport. Then, in order to correlate changes in the nanostructure of the amorphous phase with the barrier properties of polyethylene, the influence of paraffin wax content on the thermal and thermomechanical properties, free volume, density of interlamellar regions and the oxygen permeation was investigated.

## Experimental

### Materials

High density polyethylene (HDPE) Hostalen GC 7260 with density ρ = 0.960 g/cm^3^ and melt flow index (MFI) of 8 g/10 min (at 190 °C, 2.16 kg according to ISO 1133) was supplied by Lyondell Basell. A paraffin wax with M_w_ = 436.84 g/mol, melting point value (m. p.) 58–62 °C and boiling point (b. p.) at 322 °C was purchased from Sigma Aldrich (Sigma-Aldrich, Inc., St. Louis, United States) and used as the low molecular weight modifier. Hildebrand solubility parameters (δ) for HDPE and paraffin were 16.5 MPa^0.5^^30^, and 16.39 MPa^0.5^^31^, respectively.

### Sample preparation

The HDPE and paraffin wax were blended in the following composition range: 100/0, 99.5/0.5, 99/1, 97/3, 95/5, 93/7, and 86/14 wt/wt (HDPE/wax) by the melt compounding, using the PlastiCorder (Brabender, Duisburg, Germany) internal mixer for 5 min at the temperature of 190 °C and the rotor speed of 60 rpm. Finally, the films with thickness of 0.5 and 1 mm were prepared by compression molding (90 MPa) for 5 min in a hydraulic press at 190 °C, followed by quenching between metal blocks. The sample of pristine HDPE was also processed under the same conditions to obtain a reference material.

### Characterization

The modifier content in the HDPE/paraffin wax films was evaluated based on measurements by thermogravimetric analysis carried out with a nitrogen purge at 40 ml/min using a TGA 5500 apparatus (TA Instruments). For this purpose, the samples were heated to 300 °C at a rate of 20 °C/min and held for 120 min until the modifier evaporated from the polymer system. The content of the modifier was quantitatively estimated for each film from the difference between the mass loss of pristine HDPE and HDPE/wax samples.

The thermal behavior was determined with the use of an indium calibrated differential scanning calorimeter Q20 (TA Instruments, New Castle, DE, USA). Samples at about 6–7 mg were sealed in aluminum pans and heated at a constant rate of 10 °C/min in nitrogen atmosphere with an empty pan as the reference probe.

In order to correctly calculate the crystallinity of the HDPE matrix in blends, the weight of the introduced wax (based on the TGA analysis) was subtracted from the sample weight. The equation to calculate the crystallinity was as follows: *X*_*c*_ = Δ*H*_*m*_*/ΔH*_*m*_^0^ × 100, where Δ*H*_*m*_ was the sample enthalpy of fusion, Δ*H*_*m*_^0^ the enthalpy of fusion of HDPE crystal (293 J/g)^32^.

A wide-angle X-ray scattering (WAXS) measurements were performed on a computer-controlled goniometer coupled with a CuKα radiation source (λ = 0.154 nm) at 30 kV and 50 mA (Panalytical B.V., Almelo, The Netherlands). The diffraction data from the crystallographic plane (002) of orthorhombic form of polyethylene (diffraction maximum centered around 2Θ = 74.47°) were used to determine the crystallite length in the normal direction to this plane by means of the Scherrer equation:

1 $$L_{hkl} = \frac{0.9 \times \lambda}{{ \beta\times cos\Theta }}$$

where *L*_*hkl*_ is a crystallite length in the direction perpendicular to (hkl) plane, λ is X-ray wavelength, β is the half-width of a diffraction peak, and Θ is the Braggs diffraction angle. Other details of the calculations can be found in the previous report^33^.

The lamellar structure of analyzed samples was probed with use of 2-dimensional small angle X-ray scattering (SAXS). Technical details of the measurement method and long period calculations were presented elsewhere^34^.

Dynamic thermomechanical analysis was performed in a single cantilever bending mode by using a Q-800 analyzer (TA Instruments, New Castle, DE, USA). The dynamic temperature sweeps were conducted at constant frequency equal to 1 Hz within temperature scan ranging from − 130 to 100 °C under a fixed deformation of 0.02% (heating rate of 2 °C/min). In addition, dynamic mechanical tests were carried out for the selected material at different frequencies, e.g. 0.1, 10 Hz and at a heating rate of 0.2 °C/min.

The morphology analysis of the cross sections was done on freeze-fractured samples using a scanning electron microscope (SEM), Jeol JSM 5500LV model (Tokyo, Japan). The fracture surfaces were sputtered with a thin layer of gold (approx. 20 nm) before observation.

Density was measured using a gradient column (DCG) constructed from a isopropanol and water solution in accordance with ASTM D 1505^35^. All the measurements were at 23 °C to prevent temperature-dependent density changes. Glass floats were used as calibration beads in the column to determine the local density. The details of the sample density (ρ) and density of the amorphous phase (ρ_a_) calculations were described elsewhere^28^. The density of crystalline polyethylene (ρ_c_) of 1.00 g/cm^3^ was used in the calculations of amorphous density^36^.

The positron annihilation lifetime spectroscopy (PALS) experiments were conducted in air at room temperature using an ORTEC “fast–fast” nuclear spectrometer with a time resolution of about 300 ps (full width at half maximum)^37, 38^. The measurement method details was described in previous paper^39^. ^22^Na radioactive isotope was used as the source of positrons and was placed between two identical samples that were 1 mm thick. The mean positron implantation depth (L) in the material was estimated on the basis of the Eq. (4) from^40^. Taking the atomic number Z values in the range of 2.25–5.73^41–43^ and density ρ = 0.96 g/cm^3^ one can obtain L, which is in the range of 275–326 µm. These values are more than three times lower than the thickness of the samples subjected to PALS analysis.

The obtained PALS spectra were fitted by employing three exponential decays and the longest component was associated with ortho-positronum (o-Ps) lifetime. The o-Ps lifetime (τ_3_) can be correlated to the hole dimension and the hole radius (r) and can be estimated by means of the Tao-Eldrup model following the equation:

2 $$\tau_{3} = \frac{1}{{\lambda_{3} }} = 0.5\left[ {1 - \frac{r}{r + \Delta r} + \frac{1}{2\pi }\sin \left( {\frac{2\pi r}{{r + \Delta r}}} \right)} \right]^{ - 1}$$

where τ_3_ is given in nanoseconds. The factor of 0.5 ns is the inverse of the spin-averaged Ps annihilation and Δr = 0.166 nm, represents the depth of the penetration of the Ps-wave function into the walls of the hole^44, 45^.

By spherical cavity assumption, the fractional free volume (FFV) can be calculated from the o-Ps lifetime (τ_3_) and intensity (I_3_) according to formula^46^:

3 $$FFV = C \times \frac{{4\pi r^{3} }}{3}{ } \times I_{3}$$

where *C* is an empirically determined constant, characteristic for each material^47^. Due to difficulty of determining the value of the constant for many polymers and the fact that in the work it is important to know the evolution of FFV with increasing modifier content in HDPE/paraffin wax systems, the free volume fraction is given in relative amounts:

4 $$FFV = \left( {FFV_{{HDPE_{w} }} - FFV_{HDPE} } \right)/FFV_{HDPE}$$

where ${FFV}_{HDP{E}_{w}}$ and ${FFV}_{HDPE}$ are the fractional free volume of polyethylene/wax systems and pristine polyethylene, respectively.

The oxygen permeation rate analyzes were performed on a Lyssy Model L100-5000 (GPA, Systech Illinois) at 23 °C and with pressure difference of 0.1 MPa. The test was followed using the manometric method in accordance with PN-EN ISO 2556: 2002. All measurements were made through the polyethylene and polyethylene/wax films with a thickness of about 500 μm.

The paraffin wax from the blends with polyethylene was removed by extraction in a bath containing a (4:1:1) mixture of hexane, chloroform and ethanol. The extraction process was carried out at room temperature for 5 days, then the samples were dried and subjected to further studies.

## Results and discussion

**Figure 1:**
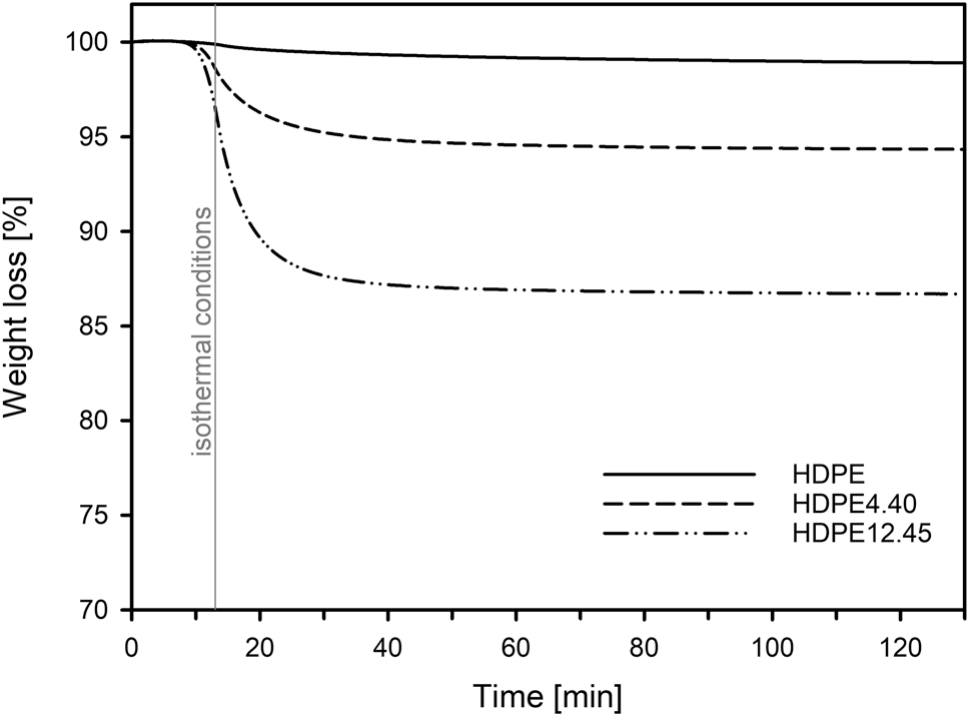
TGA weight loss curves for high density polyethylene (HDPE), polyethylene/wax system containing 4.40 and 12.45 wt% of modifier (HDPE4.40 and HDPE12.45). Samples were heated to 300 °C at a rate of 20 °C/min and held under the isothermal conditions for 120 min to evaporate the modifier from the polymer matrix.

Polyethylene and polyethylene/modifier systems with the following content (wt%) of paraffin wax: 0 (HDPE), 0.40 (HDPE0.40), 0.85 (HDPE0.85), 2.50 (HDPE2.50), 4.40 (HDPE4.40), 6.35 (HDPE6.35), and 12.45 (HDPE12.45) (determined by TGA in isothermal conditions, Fig. 1 and Table 1), were obtained by process of melt blending and analyzed in the context of highlighting the correlation between the nanostructure of the amorphous phase and barrier properties. Worth it notice that due to the similar chemical structures and close Hildebrand solubility parameter, HDPE has a good compatibility with paraffin wax. The difference of the solubility parameters (δ_polymer_–δ_modifier_)^2^ is only 0.012.

**Table 1:** Selected thermal and structural properties of the polyethylene and polyethylene/wax systems, ^*^by TGA, ^**^by DSC, and ^***^by SAXS.

	Paraffin wax content [wt%]*	Melting temperature [°C]**	Crystalline mass fraction [%]**	Long period (LP) [nm]***
HDPE	0	132.6 ± 1.6	67.0 ± 1.0	21.3 ± 0.4
HDPE0.40	0.40 ± 0.02	132.4 ± 1.6	66.8 ± 1.1	21.4 ± 0.5
HDPE0.85	0.85 ± 0.02	132.2 ± 1.4	66.7 ± 0.9	21.6 ± 0.4
HDPE2.50	2.50 ± 0.04	132.3 ± 1.5	67.1 ± 1.2	21.8 ± 0.5
HDPE4.40	4.40 ± 0.07	131.3 ± 1.3	66.6 ± 0.9	22.1 ± 0.3
HDPE6.35	6.35 ± 0.09	130.3 ± 1.3	66.8 ± 1.0	22.3 ± 0.6
HDPE12.45	12.45 ± 0.19	128.9 ± 1.2	66.6 ± 1.0	22.4 ± 0.4

**Figure 2:**
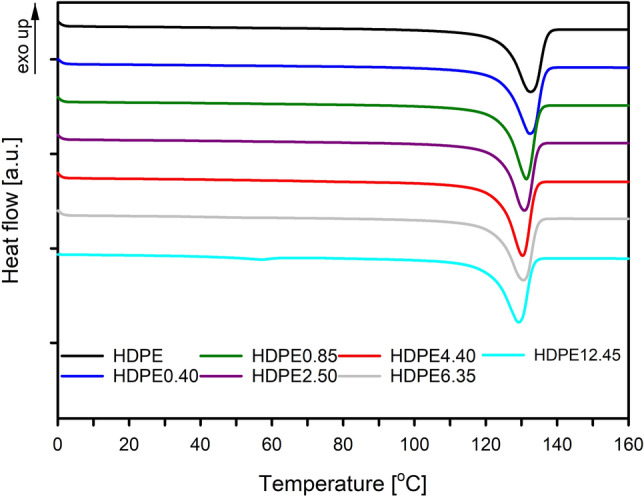
DSC thermograms of high density of polyethylene (HDPE) and polyethylene with different contents of paraffin wax (HDPE/wax). Thermograms have been shifted along the vertical axis for better visualization.

**Figure 3:**
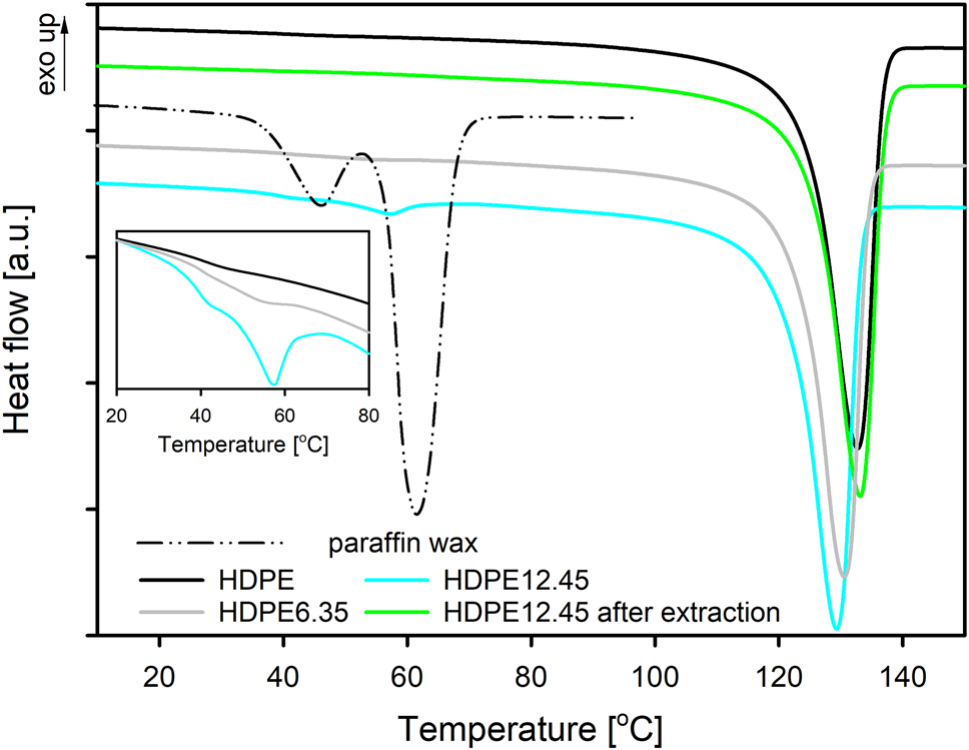
DSC thermograms of high density polyethylene (HDPE), polyethylene/wax system containing 6.35 and 12.45 wt% of modifier (HDPE6.35 and HDPE12.45) and polyethylene/modifier system after modifier removal (HDPE12.45 after extraction).

Consistent with our previous research^27^, a large, single endothermic melting peak could be recognized in the melting curves of all HDPE systems (Fig. 2). For HDPE systems with a wax content of 6.35 and 12.45%, additional small peaks were noted in the DSC curves. As can be seen from the curves presented in Fig. 3, they correspond to the melting temperature of pure (separated) paraffin, which will be discussed later in the manuscript.

The melting temperature (*T*_*m*_) of crystals for pristine HDPE equals 132.6 °C, and its melting temperature slightly shifts towards lower values after incorporating molecules of wax (Table 1, Fig. 2). This effect is probably not induced by the decrease of the crystals thickness. From the literature data based on the equilibrium thermodynamic phase diagrams of polyethylene/diluent, it is known that the addition of a diluent lowers the chemical potential of binary blends and depresses the equilibrium melting point of HDPE^48, 49^. Consequently, the depression on the melting temperature is also observed, even in the case of crystals with identical thickness.

**Figure 4:**
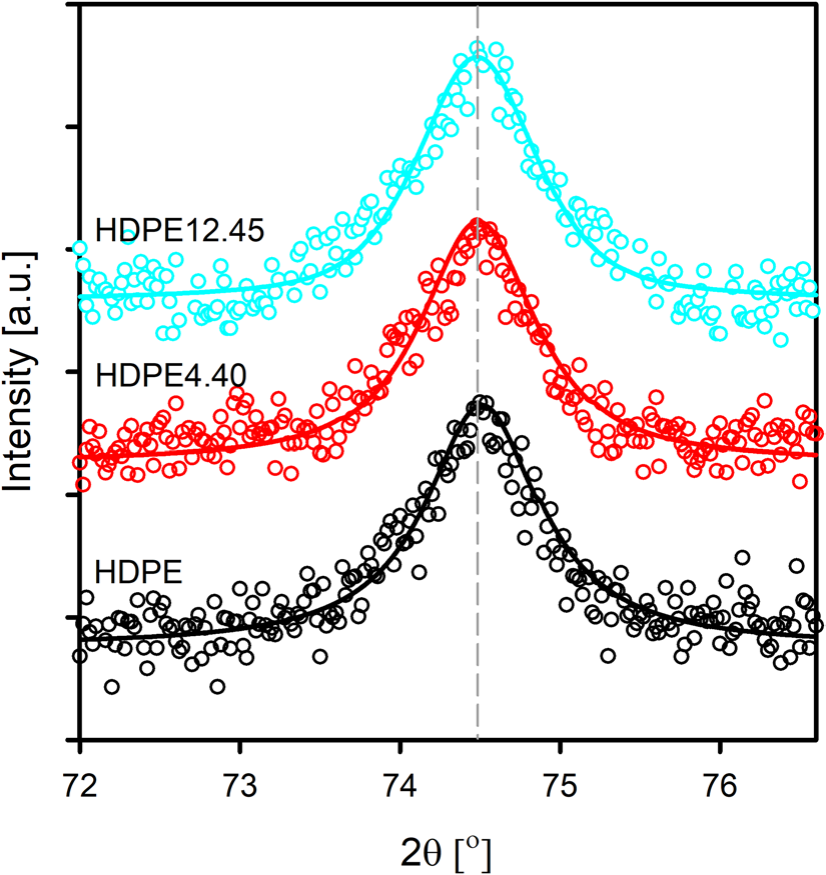
WAXS patterns of pristine HDPE, and its selected blends with paraffin wax. The experimental data and smoothing curves were shifted along the vertical axis for better visualization.

To confirm that the crystal structure of the polyethylene matrix does not change under the influence of paraffin wax molecules, measurements using the wide-angle X-ray scattering technique have been performed. Figure 4 shows WAXS patterns of the reflection located at about 2θ = 74.47° for samples of pristine HDPE and selected HDPE/wax systems (HDPE4.40 and HDPE12.45).

The intensity and position of the diffraction peak at 74.47°, indexed in the literature as the (002) crystallographic plane of HDPE, remain unchanged. The analysis of this peak allows to obtain information not only about a possible change of interplanar distance in the chain direction as a result of the introduction of wax, but also to determine, on the basis of the Scherrer Eq. (1), the length of crystallites in normal direction towards the surface of the lamellae (which is equal with the thickness of lamellar crystals). In order to evaluate the half width of the selected peak, the process of deconvolution of the collected diffraction patterns was carried out using the WAXSFit software^50, 51^. The crystallite length (length of the undisturbed crystal fragment) calculated in the direction normal to the (002) plane for pristine HDPE was 13.68 nm. This value is similar to the value of crystal thickness (13.42 nm) determined from the long period (21.3 nm, Table 1) and crystalline volume fraction (63%, by transforming the crystalline mass fraction, 67%, with the use of densities of crystalline (*d*_*c*_) and amorphous (*d*_*a*_) component: *d*_*c*_ = 1.000 g/cm^3^, *d*_*a*_ = 0.850 g/cm^3^). Lamellar crystal thickness estimated using the Scherrer equation for samples containing 4.40 and 12.45 wt% paraffin wax was 13.72 and 13.69 nm, respectively. The lack of change in the thickness of the crystals unquestionably proves that the present molecules of wax leave the structure of the HDPE crystalline component unchanged, and therefore do not show the ability to cocrystallize with the polymer matrix. These results are consistent with the results of work devoted to blends of high-density polyethylene with different waxes (hard and soft), in which the authors also did not observe the effect of cocrystallization of the wax molecules with the HDPE chains^52^. Gumede et al.^53^, based on additional, successive self-nucleation and annealing (SSA) measurements, explained that only lamellae formed by the linear segments of the linear low-density polyethylene chains with the highest short chain branch contents are able to cocrystallize with wax chains of similar lengths.

The lack of influence of the low molecular weight modifier on the microstructure of the crystalline component of polymer was also observed in other systems: polypropylene/nonadecane (*N*), polyethylene/*N*, polylactide/triethyl citrate (TEC) and aliphatic–aromatic copolyester/TEC^54^. In all these mentioned polymer/modifier systems, after removing the modifier, the melting peak temperature shifted to the value characteristic for a given pristine polymer matrix. A similar situation occurs in the case of HDPE/wax systems. As shown in Fig. 3, the removal of the introduced wax shifts back the maximum of melting peak to a value characteristic of pristine polyethylene, which clearly indicates that the microstructure of the crystalline phase in the pure polymer and its blends is the same. This finding shows explicitly that the modifier introduced into the polyethylene matrix penetrates into it and accumulates in the amorphous phase of the polymer, and therefore does not change the thickness of the crystals. Sotomayor et al.^49^ also clarified that the paraffin wax is trapped in the inter-lamellar region of HDPE, when the content of wax is lower than 20%.

The presence of modifier in HDPE systems does not also affect the crystallinity of the polyethylene matrix. As can be seen from the data in Table 1, the degree of crystallinity of all analyzed systems ranges from 66 to 67%. That constant value of the degree of crystallinity for systems containing various amounts of wax once again confirms that the cocrystallization of the wax molecules with HDPE chains is highly unlikely. The lack of changes in crystallinity and the absence of characteristic endothermic peaks for wax in the thermograms collected for polyethylene systems with wax content up to 4.40 wt% denotes that the modifier is located in the amorphous regions of the polymer and is not able to crystallize inside the polymer matrix. The wax specific signals, however, are visible in the thermograms of the HDPE6.35 and HDPE12.45 systems (Fig. 3). Although the magnitude of the melting peaks of the wax crystals is inconsiderable, their mere presence is indicative of modifier phase separation. Taking the abovementioned into account the molecules of modifier in the case of systems containing paraffin wax up to 4.40 wt% are dispersed in the material at the molecular level, in non-crystalline regions. In the remaining systems, as a result of phase separation phenomenon, modifier-rich domains are formed, the presence of which may be of key importance for the final barrier properties.

**Figure 5:**
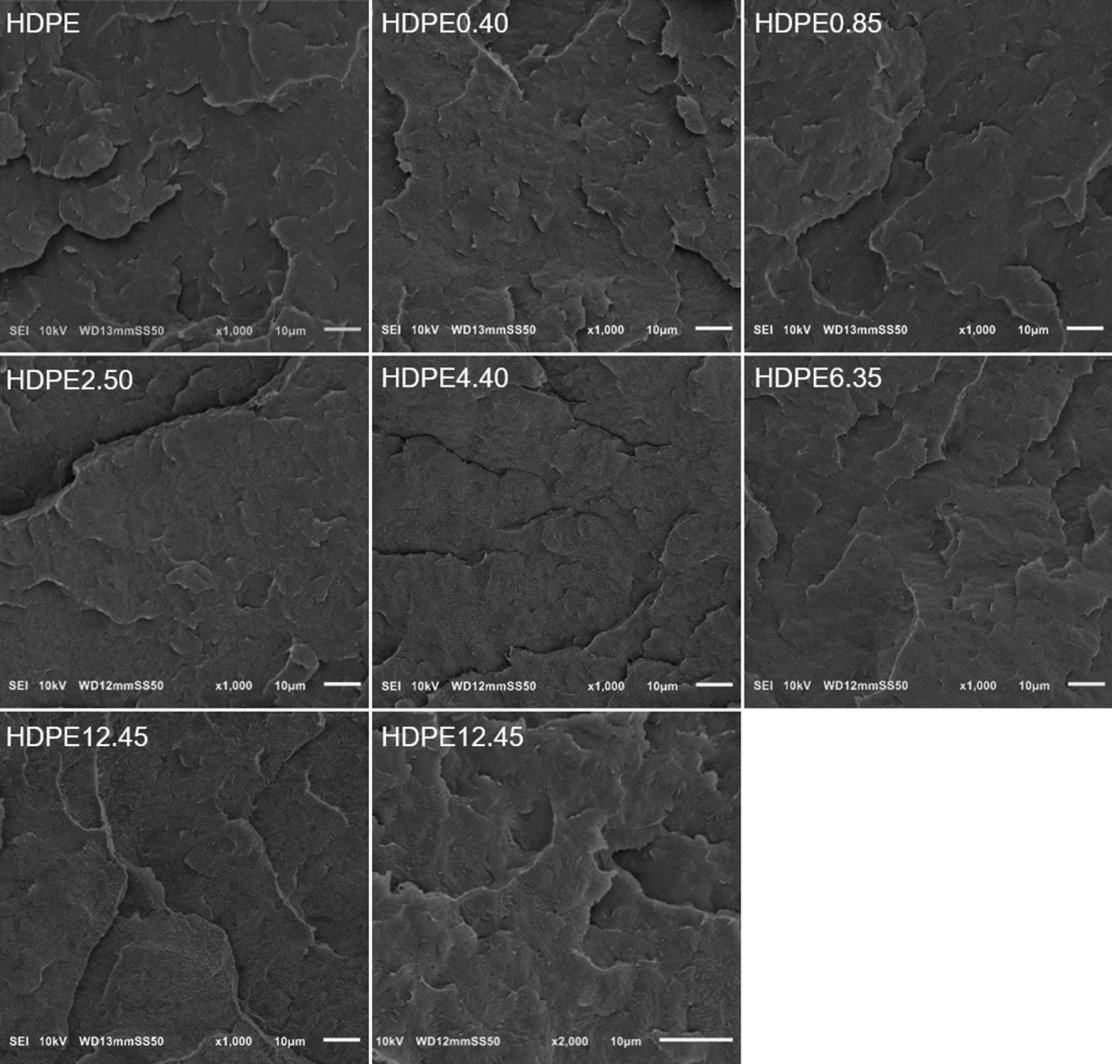
Cryo-fractured surfaces of HDPE and HDPE/paraffin wax system films.

Scanning electron microscopy (SEM) employed to further evaluate phase morphology of polyethylene and polyethylene/wax systems confirm good dispersion of the wax in the polymer matrix. In the SEM images presented in Fig. 5, it is evident that after wax incorporation, the film cryo-fractured surface of HDPE/wax systems retains the typical brittle fracture morphology analogous to that of pristine polyethylene. A fairly homogeneous surface, without distinguishable modifier inclusions, air gaps or cracks, is observable for all samples of the HDPE/wax system, even those systems where, as shown by the DSC results, there is phase separation and modifier-rich domains are present. Separated wax domains are difficult to find in SEM images recorded for systems containing 6.35 and 12.45 wt% paraffin wax. It can therefore be assumed that modifier-rich domains are likely to be smaller than 0.5 μm.

The small angle X-ray scattering (SAXS) data from which the long period (LP) was determined (Table 1) shows that LP of HDPE system samples expands upon incorporation of the wax molecules. The increase in the LP value with the content of wax in the polymer matrix confirms that the modifier, due to its location preference, contributes to the swelling of the interlamellar regions. This effect is distinctly visible for systems containing paraffin wax in the amount not exceeding 4.40 wt%. Further increasing the amount of wax in the blend with polyethylene results in only a slight increase in the long period.

**Figure 6:**
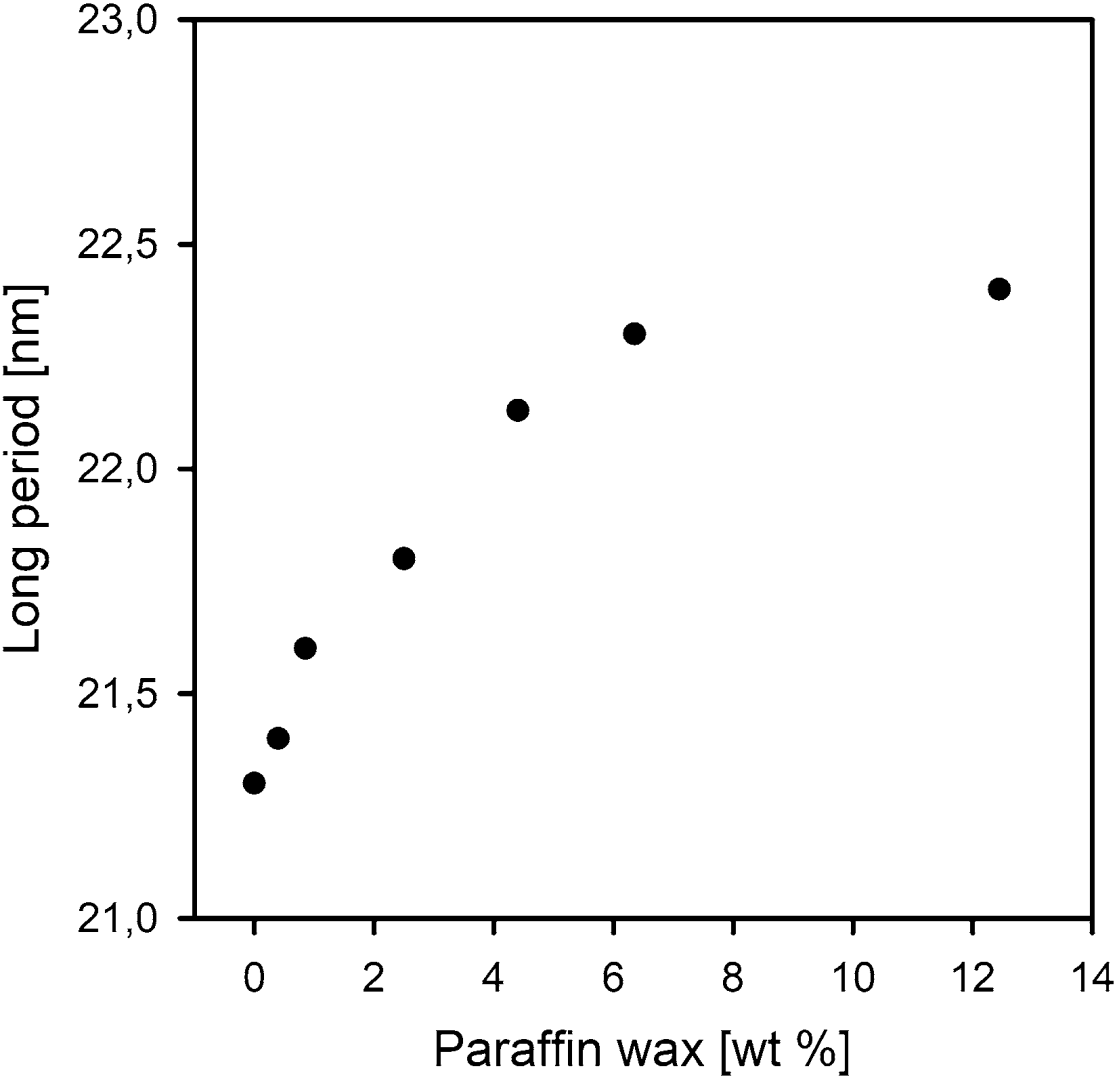
The long period values plotted against paraffin wax content in the HDPE/wax systems.

As can be seen from the Fig. 6, the curve of changes of the long period as a function of wax content starts to reach a plateau at wax content above 6 wt %, what proves the phase separation phenomenon for these systems.

With the above in mind, it is worth answering the question: How does the degree of swelling of the amorphous phase, depending on the content of the modifier, affect the molecular packing density of HDPE and, thus, the content of free volume/dimension of free volume pores i.e., key parameters in terms of barrier properties?

**Figure 7:**
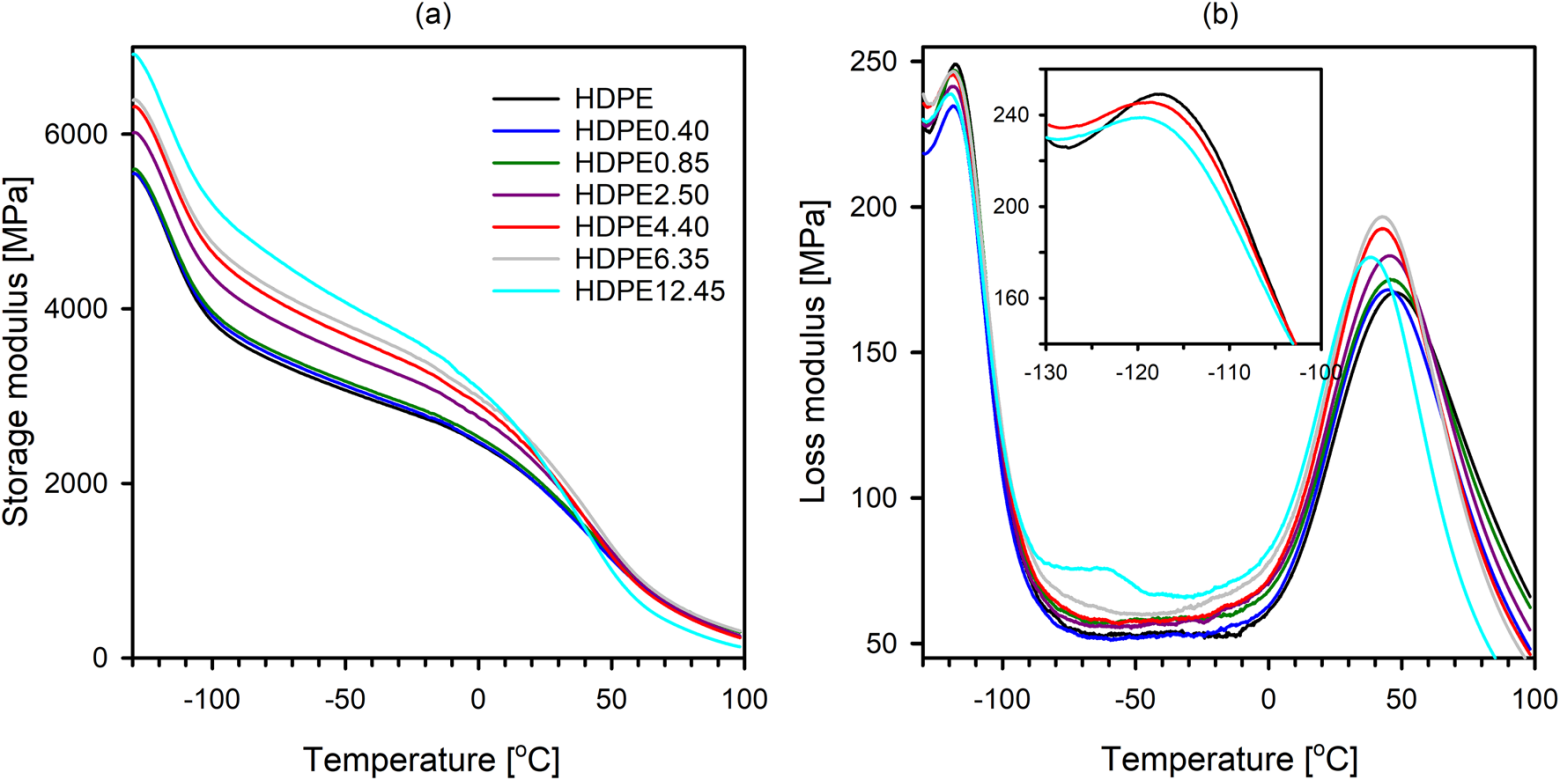
Temperature dependence of the storage (**a**) and loss (**b**) modulus of pristine and modified with paraffin wax HDPE films.

Dynamic mechanical analysis can provide information regarding characteristic parameters of polymer microstructure, including molecular density of amorphous regions and the relaxation behavior of molecular chains. Figure 7a,b show the dependence of storage modulus (E′) and loss modulus (E″) on temperature, respectively.

In Fig. 7a, the E′ values show a decreasing trend for all the systems with two decrement stages as the temperature increases. The first stage approximately at − 120 °C is ascribed to γ relaxation, and the second stage (from 40 to 100 °C) to α relaxation. The γ relaxation process is associated with small-scale motions (the motion of three or four methyl groups of the main chain) within the amorphous component of PE^55, 56^, and in literature data it is assigned to the brittle-ductile transition phenomenon^57, 58^. The α relaxation, in turn, is attributed to motions in the intercrystalline (intralamellar slip process or grain boundary phenomena) and intracrystalline regions (transitional motion of chain segments along the *c* axis within the crystal lattice)^59, 60^. Therefore, the shift of α relaxation towards lower temperatures is usually observed with a decrease of crystal thickness. It is worth noting that practically in the whole temperature range the values of E′ show increase with increase in wax content. Such an effect may be related to the enhanced mechanical restraints imposed by increased content of paraffin wax molecules on the molecular mobility of HDPE chains^49^. Paraffin wax (below 40 °C) immobilizes the polymer chains and thus leads to an increase in the modulus of the polyethylene matrix. In the case of system with higher wax content (HDPE12.45), above the wax melting temperature, a decrease in modulus can be seen.

Plots of E″ against temperature (Fig. 7b) clearly show that the paraffin wax incorporated into HDPE has a measurable impact primarily on the α relaxation, but also on γ relaxation. The shift of the α relaxation temperature towards lower values and the increase in the intensity of α process evidence the improvement of molecular mobility between the intra-lamellar block grain boundary^61^. This may also reflect the presence of additional stresses within the lamellar crystals^27^, but is definitely not related to changes in the thickness of the crystals or cocrystallization phenomenon (as proved above). The decrease of the γ relaxation temperature with the increasing content of paraffin wax, visible in the inset in Fig. 7b, is associated with enhancement in the mobility of the polymer chains fragments within amorphous regions^62^.

The dynamic mechanical spectra for the system samples containing paraffin wax > 4.40 wt% show an additional relaxation peak centered at about − 60 °C. The relaxation presented in the loss modulus curves of the HDPE6.35 and HDPE12.45 samples results from the glass transition temperature of the paraffin wax-rich phase and confirms the phenomenon of phase separation in these systems. The signature of modifier phase separation becomes more pronounced as the amount of incorporated wax increases. The inclusion of paraffin wax into the HDPE matrix in an amount not exceeding 4.40 wt% does not allow for the separation of modifier-rich domains. Considering the lamellar cluster model of HDPE swelling^57^, the incorporated molecules of wax are likely to effectively fill the empty spaces (free volume pores) between the chains in the interlamellar amorphous phase.

In addition to the chain dynamics, the molecular packing/density of amorphous regions and the fractional free volume were assessed to monitor the impact of paraffin wax molecules on the nano-/microstructure of amorphous regions of HDPE.

**Figure 8:**
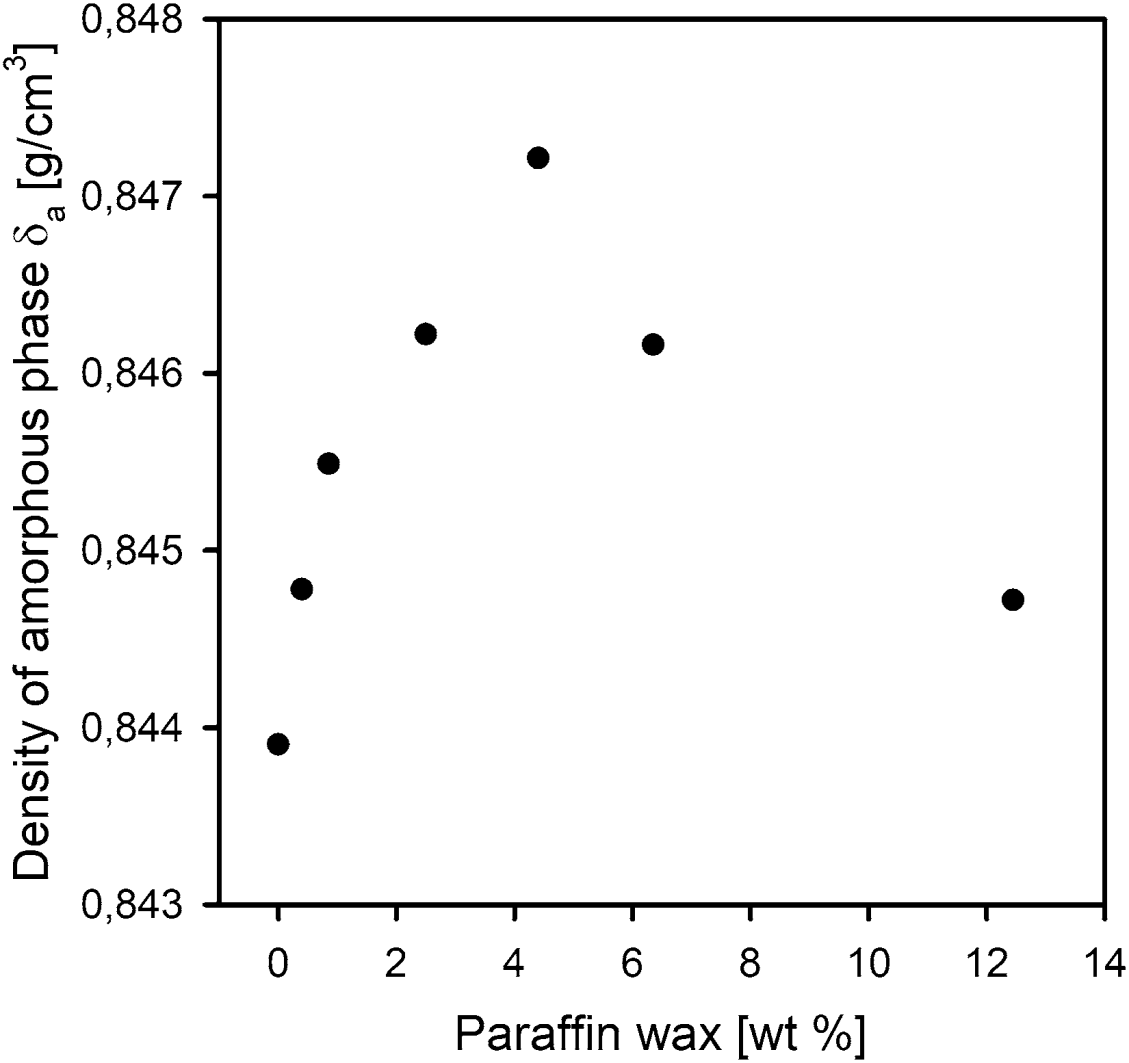
Density of amorphous phase for pristine HDPE and HDPE/wax system samples.

The evaluation of changes in the packing density of the amorphous phase under the influence of the introduced wax molecules was carried out using a density gradient column (DGC). The density of the amorphous regions was calculated using overall density from DGC and presented in Fig. 8.

The results show an increase in density with an increase in the wax content, but only for HDPE blends that contain modifier in an amount not exceeding 4.40 wt%. A further increase in the amount of wax molecules in the HDPE matrix results in a decrease in the amorphous density, but still remains higher than that of pristine HDPE. The densification observed for all HDPE/wax systems it can be induced by the improved molecular packing of the amorphous regions^63^. Due to the fact that the molecular packing is closely related to the amount of free volume, it seems that the HDPE4.40 system with the highest density of the amorphous phase should be characterized by the lowest free volume content. With the foregoing in mind, it should be stated that the inclusion of the modifier alters the molecular packing and, thus, the free volume of the interlamellar amorphous regions.

To verify the molecular packing/the content of free volume of the interlamellar amorphous regions—a parameter that determines the final barrier properties, PALS analysis was performed for selected HDPE systems. The mean o-Ps lifetime (τ_3_) connected with the average size of the free volume pores of the amorphous phase and the corresponding intensity (I_3_) proportional to the number of free volumes holes were analyzed.

**Figure 9:**
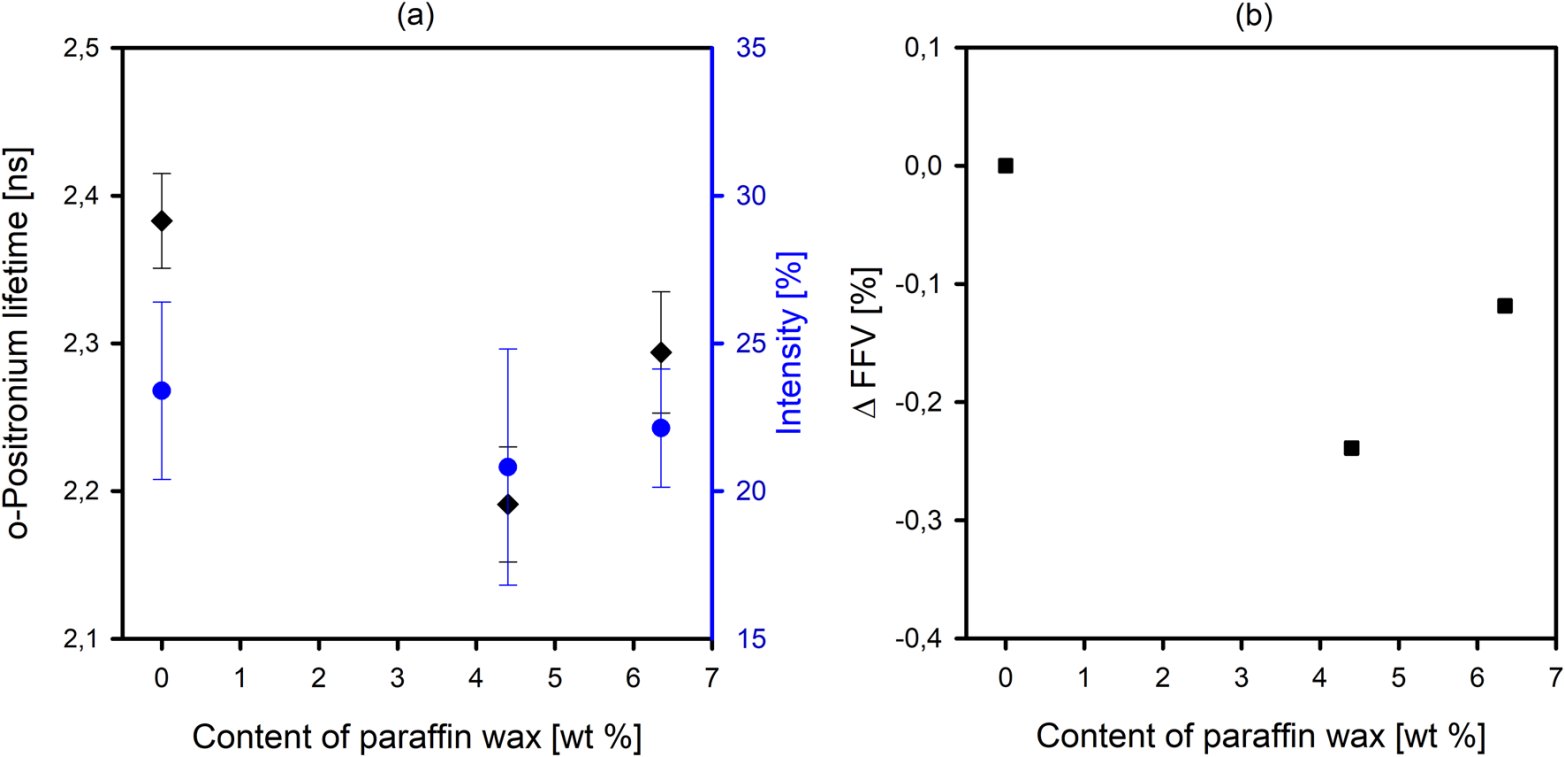
Variation of mean size and number of free volume pores (**a**), and free volume fraction (**b**) as a function of wax content.

Figure 9 illustrates that the addition of wax molecules to HDPE matrix reduces both the τ_3_ value and the I_3_ value. The trend of changes in the size and number of the free volume pores in the amorphous regions of HDPE due to the incorporation of wax molecules is analogous to that observed in density measurements. The fractional free volume ΔFFV (Fig. 9b), shows a downward trend compared to pristine HDPE, what corresponds very well with the measurements of the density of amorphous regions. The increase in the value of the ΔFFV parameter in the case of a blend containing 6.35 wt% wax compared to the HDPE4.40 system is probably associated with the phenomenon of voids formation at the interphase between the polymer matrix and the separated wax.

The abovementioned results exhibit that the inclusion of paraffin wax in HDPE systems changes the molecular packing in the interlamellar amorphous regions affecting the free volume fraction, which should be reflected in the barrier properties. Transport properties of O_2_ molecules through HDPE blend systems were determined by permeation measurement in order to recognize the effect of polyethylene/wax composition on the final barrier properties. It is worth remembering that the content of the modifier, its compatibility with the polymer matrix and dispersion are critical factors that can affect the permeability of polymer blends.

**Figure 10:**
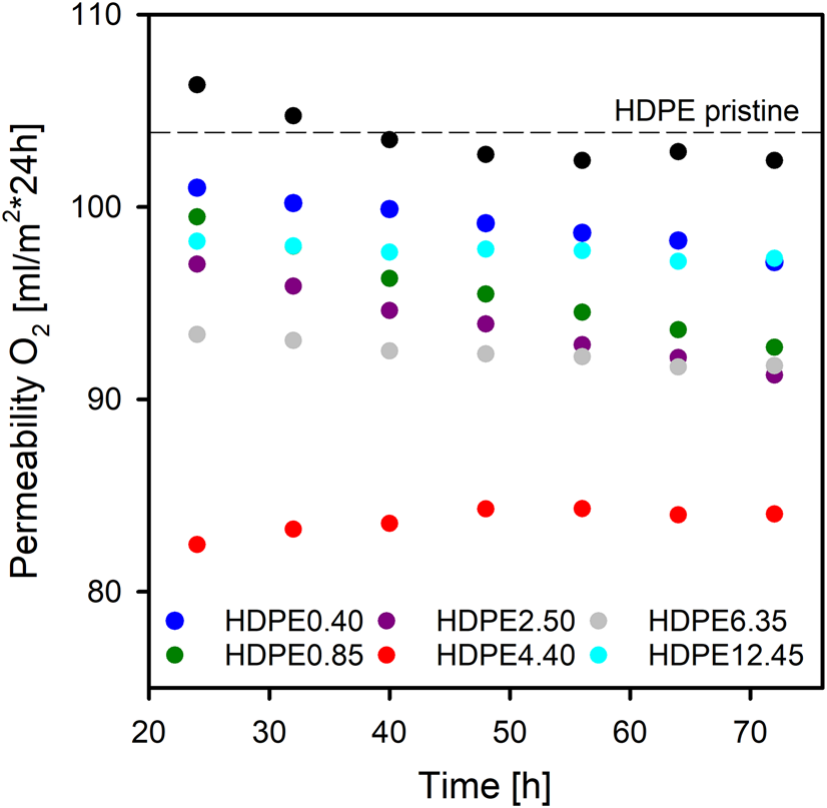
The oxygen permeability of HDPE and its blends with paraffin wax as a function of time that has passed since the film was prepared.

The barrier properties of the analyzed systems insensibly change over time (Fig. 10). This phenomenon is correlated with generally understood aging/balance processes initiated in samples after their solidification. A general observation from Fig. 10 is that the addition of wax molecules enhanced the barrier properties of HDPE to oxygen. The measured oxygen permeability for pristine polyethylene is 104 mL/m^2^*24 h. Interestingly, the introduction of paraffin wax into the polymer matrix, regardless of its content in the blend with polyethylene, results in a decrease in the permeability value. It is worth noting that the oxygen permeability of the analyzed systems decreases with the increase of the modifier content until the paraffin content in the HDPE/paraffin wax systems reaches 4.40 wt%. Further increasing the amount of wax in the blends with polyethylene causes a slight deterioration of the barrier properties, and thus an increase in the volume of O_2_ permeable through the sample, however, as mentioned earlier, systems containing 6.35 wt% (or more) of paraffin wax, compared to the reference system, show an improvement in the barrier properties. The filling effect of wax molecules in the matrix of HDPE, which hinders the oxygen transmission by molecular “densification” of the amorphous regions, results in an improved barrier properties. In the case of the HDPE6.35 and HDPE12.45 systems, apart from the densification of amorphous areas under the influence of the incorporated modifier, phase separation is observed. The presence of modifier-rich domains in these systems, as mentioned above, promotes the formation of an interphase between the polymer matrix and the separated wax, facilitating the transport of oxygen molecules.

The lowest permeability, and thus the greatest increase in the oxygen barrier, amounting to 22%, is characterized by the HDPE/wax system containing 4.40 wt% of paraffin wax, which among all the analyzed systems has the most “dense” amorphous phase with a density of 0.8472 g/cm^3^ (the amorphous phase density of pristine polyethylene δ_a_ = 0.8439 g/cm^3^). This effective increase in the packing/molecular density of the amorphous polyethylene phase, despite increasing interlamellar distance (Table 1, Fig. 6), is possible because the changes occurring at the free volume level are compensated by the presence of wax molecules. The incorporated molecules of wax display the ability to fill empty spaces (free volume pores) between the chains in the interlamellar amorphous phase.

Interestingly and noteworthy is the fact that based on the trend of changes in the density of the amorphous phase of polyethylene (determined by the density gradient column method) as a function of the paraffin wax content, it is possible to plot the trend of oxygen permeability for these systems—and vice versa.

## Conclusions

The parameters of oxygen permeability through the HDPE/paraffin wax systems were analyzed with the aim to understand the impact of the molecular packing efficiency of the amorphous phase (increase or decrease in the density of amorphous regions consisted with decrease or increase in the pore size of the free volume) on the overall transport properties of semicrystalline polyethylene. By introducing small amounts of low molecular weight modifier, which penetrates and accumulates in the interlamellar regions, it was possible to differentiate the packing density of the amorphous regions of HDPE. Density measurements revealed that the incorporation of paraffin wax into the polyethylene matrix leads to an increase of the density of the blends stimulated mainly by an increase of the density of amorphous regions. Importantly, polyethylenes with “densified” amorphous regions showed improved barrier properties compared to the reference material, with no apparent changes in thermal and thermomechanical properties. It is worth noting that the degree of densification depended on the swelling degree of the amorphous regions and the size of the change of interlamellar distance (the size of long period). The HDPE4.40 system film, with amorphous density increased by 0.003 g/cm^3^ compared to pristine HDPE, displayed an almost 22% reduction in oxygen permeability. The tendency of changes in the amorphous phase density depending on the wax content is analogous to changes in the fractional free volume, namely the increase in the packing density of interlamellar areas was accompanied by a decrease in the free volume pore size. The above clearly confirms the validity of using density measurements in defining the role of amorphous layer nanostructure in the barrier properties of semicrystalline polymers. The molecular packing efficiency of the amorphous phase in semicrystalline polymer blends, which is determined by the interactions between the modifier molecules and the polymer matrix, including the solubility parameters, is of fundamental importance in the context of gain the optimal barrier properties.

## Data Availability

The datasets used and/or analyzed during the current study will be made available from the corresponding author on reasonable request.

## References

[1] Wu F, Misra M, Mohanty AK (2021). Challenges and new opportunities on barrier performance of biodegradable polymers for sustainable packaging. Prog. Polym. Sci..

[2] Tsakona, M. & Rucevska, I. Baseline report on plastic waste. document No. UNEP/CHW/PWPWG.1/INF/4: basel convention. 68 (United Nations Environment Programme (UNEP), 2020).

[3] Noh MICM, Mohamed MA, Ismail AG, Ani MH, Majlis BY (2019). Improvement of gas barrier properties of polyethylene terephtalate (PET) by graphene nanoplatelets (GNP). Mater. Today Proc..

[4] Youssef AM, El-Sayed SM (2018). Bionanocomposites materials for food packaging applications: Concepts and future outlook. Carbohyd. Polym..

[5] Xie FW, Pollet E, Halley PJ, Averous L (2013). Starch-based nano-biocomposites. Prog. Polym. Sci..

[6] El Miri N (2015). Bio-nanocomposite films reinforced with cellulose nanocrystals: Rheology of film-forming solutions, transparency, water vapor barrier and tensile properties of films. Carbohyd. Polym..

[7] Ghaani M, Cozzolino CA, Castelli G, Farris S (2016). An overview of the intelligent packaging technologies in the food sector. Trends Food Sci. Tech..

[8] Zia J, Paul UC, Heredia-Guerrero JA, Athanassiou A, Fragouli D (2019). Low-density polyethylene/curcumin melt extruded composites with enhanced water vapor barrier and antioxidant properties for active food packaging. Polymer.

[9] Wen YH (2020). Evaluating distillers grains as bio-fillers for high-density polyethylene. J. Polym. Res..

[10] Ma ZL (2021). Thermal properties and barrier performance of antibacterial high-density polyethylene reinforced with carboxyl graphene-grafted modified high-density polyethylene. Ind. Eng. Chem. Res..

[11] Liu GJ (2021). Ultra-high gas barrier composites with aligned graphene flakes and polyethylene molecules for high-pressure gas storage tanks. J. Energy Storage.

[12] Hemavathi AB, Babu A, Murthy PSK, Zarena AS (2022). Sustainable nanocomposite films of improved barrier properties for food packaging applications. Mater. Today Proc..

[13] Kurek M, Scetar M, Voilley A, Galic K, Debeaufort F (2012). Barrier properties of chitosan coated polyethylene. J. Membr. Sci..

[14] Sahi S, Djidjelli H, Boukerrou A (2021). Study of the properties and biodegradability of the native and plasticized corn flour-filled low density polyethylene composites for food packaging applications. Mater. Today Proc..

[15] Lozay Q (2021). Structural and barrier properties of compatibilized PE/PA6 multinanolayer films. Membr. Basel.

[16] Golebiewski J, Rozanski A, Dzwonkowski J, Galeski A (2008). Low density polyethylene-montmorillonite nanocomposites for film blowing. Eur. Polym. J..

[17] Zenkiewicz M, Richert J, Rozanski A (2010). Effect of blow moulding ratio on barrier properties of polylactide nanocomposite films. Polym. Test..

[18] Harton SE (2010). Immobilized polymer layers on spherical nanoparticles. Macromolecules.

[19] Wu SY (2015). Aligning multilayer graphene flakes with an external electric field to improve multifunctional properties of epoxy nanocomposites. Carbon.

[20] Wang H (2020). Ordered proton channels constructed from deoxyribonucleic acid-functionalized graphene oxide for proton exchange membranes via electrostatic layer-by-layer deposition. Int. J. Hydrog. Energ.

[21] George SC, Thomas S (2001). Transport phenomena through polymeric systems. Prog. Polym. Sci..

[22] Neway B, Hedenqvist MS, Gedde UW (2003). Effect of thermal history on free volume and transport properties of high molar mass polyethylene. Polymer.

[23] Kanehashi S, Kusakabe A, Sato S, Nagai K (2010). Analysis of permeability; solubility and diffusivity of carbon dioxide; oxygen; and nitrogen in crystalline and liquid crystalline polymers. J. Membrane Sci..

[24] Kanehashi S, Nagai K (2005). Analysis of dual-mode model parameters for gas sorption in glassy polymers. J. Membrane Sci..

[25] Sharma SK, Pujari PK (2017). Role of free volume characteristics of polymer matrix in bulk physical properties of polymer nanocomposites: A review of positron annihilation lifetime studies. Prog. Polym. Sci..

[26] Wypych, G. *Handbook of Plasticizers*. (ChemTech Publishing, 2004, 2012 & 2017).

[27] Krajenta A, Rozanski A, Idczak R (2016). Morphology and properties alterations in cavitating and non-cavitating high density polyethylene. Polymer.

[28] Safandowska M, Makarewicz C, Rozanski A, Idczak R (2022). Barrier properties of semicrystalline polylactide: The role of the density of the amorphous regions. Macromolecules.

[29] Rozanski A, Idczak R (2015). Influence of non-polymeric substances localized in the amorphous phase on selected properties of semicrystalline polymers. Eur. Polym. J..

[30] Brandrup J, Immergut EH, Grulke EA (1999). Polymer Handbook.

[31] Voelkel A, Fall J (2014). Solubility parameter as polarity measure for high-boiling oil products. Fuel.

[32] Righetti MC, Gazzano M, Di Lorenzo ML, Androsch R (2015). Enthalpy of melting of alpha—and alpha-crystals of poly(L-lactic acid). Eur. Polym. J..

[33] Safandowska M, Rozanski A, Galeski A (2020). Plasticization of polylactide after solidification: an effectiveness and utilization for correct interpretation of thermal properties. Polym. Basel.

[34] Goderis B, Reynaers H, Koch MHJ, Mathot VBF (1999). Use of SAXS and linear correlation functions for the determination of the crystallinity and morphology of semi-crystalline polymers. Application to linear polyethylene. J. Polym. Sci. Pol. Phys..

[35] Vol. ASTM D1505–18 (2018).

[36] Auras R, Harte B, Selke S (2004). An overview of polylactides as packaging materials. Macromol. Biosci..

[37] Coleman PG (2003). Principles and applications of positron and positronium chemistry.

[38] Kansy J (1996). Microcomputer program for analysis of positron annihilation lifetime spectra. Nucl. Instrum. Meth. A.

[39] Makarewicz C, Safandowska M, Idczak R, Rozanski A (2021). Positron annihilation lifetime spectroscopic analysis of plastic deformation of high-density polyethylene. Macromolecules.

[40] Dryzek J, Singleton D (2006). Implantation profile and linear absorption coefficients for positrons injected in solids from radioactive sources Na-22 and Ge-68\Ga-68. Nucl. Instrum. Meth. B.

[41] Ochbelagh DR (2016). Estimation of effective atomic numbers of polyethylene and coal using compton scattering. Tech. J..

[42] Kiran KU, Ravindraswami K, Eshwarappa KM, Somashekarappa HM (2015). Effective atomic number of selected construction materials using gamma backscattering technique. Ann. Nucl. Energy.

[43] Naydenov SV, Ryzhikov VD, Smith CF (2004). Direct reconstruction of the effective atomic number of materials by the method of multi-energy radiography. Nucl. Instrum. Meth. B.

[44] Tao SJ (1972). Positronium annihilation in molecular substances. J. Chem. Phys..

[45] Eldrup M, Lightbody D, Sherwood JN (1981). The temperature-dependence of positron lifetimes in solid pivalic acid. Chem. Phys..

[46] Wang YY, Nakanishi H, Jean YC, Sandreczki TC (1990). Positron-annihilation in amine-cured epoxy polymers—pressure-dependence. J. Polym. Sci. Pol. Phys..

[47] Hill AJ, Weinhold S, Stack GM, Tant MR (1996). Effect of copolymer composition on free volume and gas permeability in poly(ethylene terephthalate)-poly(1,4 cyclohexylenedimethylene terephthalate) copolyesters. Eur. Polym. J..

[48] Liu M, Liu SH, Xu ZL, Wei YM, Yang H (2016). Formation of microporous polymeric membranes via thermally induced phase separation: A review. Front. Chem. Sci. Eng..

[49] Sotomayor ME, Krupa I, Varez A, Levenfeld B (2014). Thermal and mechanical characterization of injection moulded high density polyethylene/paraffin wax blends as phase change materials. Renew. Energ..

[50] Rabiej M (2013). Application of immune and genetic algorithms to the identification of a polymer based on its X-ray diffraction curve. J. Appl. Crystallogr..

[51] Rabiej M (2014). A hybrid immune-evolutionary strategy algorithm for the analysis of the wide-angle X-ray diffraction curves of semicrystalline polymers. J. Appl. Crystallogr..

[52] Luyt AS, Brull R (2004). Investigation of polyethylene-wax blends by CRYSTAF and SEC-FTIR. Polym. Bull..

[53] Gumede TP (2016). Plasticization and cocrystallization in LLDPE/Wax blends. J. Polym. Sci. Pol. Phys..

[54] Rozanski A, Safandowska M, Krajenta A (2018). DSC/SAXS analysis of the thickness of lamellae of semicrystalline polymers-restrictions in the case of materials with swollen amorphous phase. Polym. Test..

[55] Hoffman JD, Williams G, Passagli E (1966). Analysis of alpha beta and gamma relaxations in polychlorotrifluoroethylene and polyethylene dielectric and mechanical properties. J. Polym. Sci. Pol. Sym..

[56] Crissman JM (1975). Transition from paraffinic to polymeric behavior - mechanical-properties. J. Polym. Sci. Pol. Phys..

[57] Ito A, Hioki K, Kono K, Hiejima Y, Nitta K (2020). Effects of liquid paraffin on dynamic mechanical properties of linear high-density polyethylene. Macromolecules.

[58] Pechhold W, Knauss G, Eisele U (1964). Viscoelastic behavior of linear polyethylene + of paraffin mixtures. Kolloid. Z. Z. Polym..

[59] Nakayasu H, Markovitz H, Plazek DJ (1961). The frequency and temperature dependence of the dynamic mechanical properties of a high density polyethylene. T Soc Rheol.

[60] Tanaka A, Chang EP, Delf B, Kimura I, Stein RS (1973). Rheo-optical studies of nature of alpha-mechanical loss mechanism of polyethylene. J Polym. Sci. Pol. Phys..

[61] Men YF, Rieger J, Endeler HF, Lilge D (2003). Mechanical alpha-process in polyethylene. Macromolecules.

[62] Yang F (2022). Effects of diluent content on the crystallization behavior and morphology of polyethylene membrane fabricated via thermally induced phase separation process. Polymer.

[63] Paul DR, Newman S (2012). Polymer Blends.

